# Comparison of Nanotrap^®^ Microbiome A Particles, membrane filtration, and skim milk workflows for SARS-CoV-2 concentration in wastewater

**DOI:** 10.3389/fmicb.2023.1215311

**Published:** 2023-07-05

**Authors:** Pengbo Liu, Lizheng Guo, Matthew Cavallo, Caleb Cantrell, Stephen Patrick Hilton, Anh Nguyen, Audrey Long, Jillian Dunbar, Robbie Barbero, Robert Barclay, Orlando Sablon, Marlene Wolfe, Ben Lepene, Christine Moe

**Affiliations:** ^1^Center for Global Safe Water, Sanitation, and Hygiene, Rollins School of Public Health, Emory University, Atlanta, GA, United States; ^2^Ceres Nanosciences, Inc., Manassas, VA, United States

**Keywords:** SARS-CoV-2, wastewater, Nanotrap particles, RT-qPCR, grab, Moore swab

## Abstract

**Introduction:**

Severe acute respiratory syndrome coronavirus-2 (SARS-CoV-2) RNA monitoring in wastewater has become an important tool for Coronavirus Disease 2019 (COVID-19) surveillance. Grab (quantitative) and passive samples (qualitative) are two distinct wastewater sampling methods. Although many viral concentration methods such as the usage of membrane filtration and skim milk are reported, these methods generally require large volumes of wastewater, expensive lab equipment, and laborious processes.

**Methods:**

The objectives of this study were to compare two workflows (Nanotrap^®^ Microbiome A Particles coupled with MagMax kit and membrane filtration workflows coupled with RNeasy kit) for SARS-CoV-2 recovery in grab samples and two workflows (Nanotrap^®^ Microbiome A Particles and skim milk workflows coupled with MagMax kit) for SARS-CoV-2 recovery in Moore swab samples. The Nanotrap particle workflow was initially evaluated with and without the addition of the enhancement reagent 1 (ER1) in 10 mL wastewater. RT-qPCR targeting the nucleocapsid protein was used for detecting SARS-CoV-2 RNA.

**Results:**

Adding ER1 to wastewater prior to viral concentration significantly improved viral concentration results (*P* < 0.0001) in 10 mL grab and swab samples processed by automated or manual Nanotrap workflows. SARS-CoV-2 concentrations in 10 mL grab and Moore swab samples with ER1 processed by the automated workflow as a whole showed significantly higher (*P* < 0.001) results than 150 mL grab samples using the membrane filtration workflow and 250 mL swab samples using the skim milk workflow, respectively. Spiking known genome copies (GC) of inactivated SARS-CoV-2 into 10 mL wastewater indicated that the limit of detection of the automated Nanotrap workflow was ~11.5 GC/mL using the RT-qPCR and 115 GC/mL using the digital PCR methods.

**Discussion:**

These results suggest that Nanotrap workflows could substitute the traditional membrane filtration and skim milk workflows for viral concentration without compromising the assay sensitivity. The manual workflow can be used in resource-limited areas, and the automated workflow is appropriate for large-scale COVID-19 wastewater-based surveillance.

## Introduction

The coronavirus disease 2019 (COVID-19) is caused by severe acute respiratory syndrome coronavirus-2 (SARS-CoV-2), a single-stranded RNA virus that can infect individuals who can develop illness ranging in severity from life-threatening complications to mild symptomatic or asymptomatic infections. SARS-CoV-2 is mainly transmitted among people via respiratory droplets. However, the virus is also shed in feces at high concentrations, and SARS-CoV-2 RNA titer in feces has been reported to be 10^5^ copies per gram of feces or between 10^2^ and 10^7^ genome copies per milliliter of stool suspension (Jones et al., 2020). The virus eventually enters toilets, public sewage systems, and wastewater treatment plants, which allows researchers to collect wastewater and detect virus RNA from these settings (Gibas et al., 2021; Liu et al., 2022). Since the early detection of SARS-CoV-2 in wastewater (Ahmed et al., 2020; Wu et al., 2020), monitoring for SARS-CoV-2 RNA has become a critical tool for global COVID-19 surveillance and to guide response to COVID-19 outbreaks in communities. The initial step in processing wastewater samples for SARS-CoV-2 detection is usually to concentrate viruses from a relatively large volume to a small volume of pellets that can be used for nucleic acid extraction. Previous studies reported that the typical volumes of wastewater samples for the detection of SARS-CoV-2 range from 50 to 250 ml (Khan et al., 2021). For this range of volumes, SARS-CoV-2 was concentrated using several approaches, e.g., membrane filtration (Ahmed et al., 2020), polyethylene glycol (PEG) (Wu et al., 2021), skim milk (Philo et al., 2022b), ultracentrifugation (Wurtzer et al., 2020), and ultrafiltration (Fores et al., 2021). For instance, PEG and skim milk are both based on the mechanism of precipitation of viral protein. These two methods usually require a centrifuge that can spin down at least a 50-ml tube, which is a problem for some laboratories with limited resources, especially in developing countries. Using a small volume of wastewater without this centrifuge and without compromising assay sensitivity would be preferable for the broad application of SARS-CoV-2 wastewater surveillance. In addition, there are limited reports on the application of novel viral concentration approach in wastewater and consideration of small sample volume with better sensitivity.

Nanotrap particles are highly porous hydrogel particles that are versatile in their functionality for the capture and concentration of analytes, such as proteins, peptides, nucleic acids, hormones, viral antigens, and live infectious pathogens. This versatility allows Nanotrap particles to be customized to capture analytes in various complex biological mixtures such as blood, saliva, urine, and wastewater (Jaworski et al., 2014). Nanotrap particles can perform three essential functions as follows: capturing target analytes from complex metrics, separating analytes from interfering materials, and protecting target analytes from degradation. Nanotrap particles are capable of preserving Rift Valley fever viruses and RNA from degradation after sample concentration at both ambient and 37°C temperatures for up to 3 days (Shafagati et al., 2013). In addition to these advantages, Nanotrap particles can capture and concentrate multiple pathogens including viruses, bacteria, and protozoan parasites in one sample (Lin et al., 2020). This capability has been demonstrated with Nanotrap particles used in a coinfection scenario with HIV and Rift Valley fever virus in bovine serum (Shafagati et al., 2013). Since the COVID-19 pandemic, Nanotrap particles have been used to detect SARS-CoV-2 (Karthikeyan et al., 2021a,b; Zhan et al., 2022; Ahmed et al., 2023) and other respiratory viruses (Shafagati et al., 2016; Anderson et al., 2021) in wastewater. For example, Anderson et al. (2021) showed that Nanotrap particles can be used for the concentration and detection of SARS-CoV-2, influenza, and respiratory syncytial viruses in wastewater simultaneously.

Three sampling methods are commonly used for SARS-CoV-2 wastewater-based epidemiology as follows: grab, composite, and passive sampling (Rafiee et al., 2021; Augusto et al., 2022; Bivins et al., 2022). Grab sampling is a simple and convenient method where wastewater is collected at one point in time; however, interpretation of the results is limited because the samples only represent a snapshot at one moment. Composite sampling is considered a more representative method due to its ability to collect individual samples at regular time intervals, and the individual samples are subsequently combined in proportion to the wastewater flow rate. However, composite sampling is costly and time-consuming, and it may not be feasible under certain environmental conditions. Passive sampling, e.g., Moore swab, involves a material being deployed in wastewater to trap SARS-CoV-2 over time, and it provides a sensitive, low-cost, and convenient alternative to composite sampling.

The objectives of this study were to (a) compare SARS-CoV-2 detection in 10 ml of wastewater with and without enhancement reagent for both automated and manual Nanotrap workflows, (b) compare SARS-CoV-2 detection in 10 ml of wastewater using automatic Nanotrap concentration workflow and in 150 ml of wastewater using membrane filtration workflow as a whole, (c) compare SARS-CoV-2 detection in 10 ml of Moore swab samples using automated Nanotrap workflow and in 250 ml of Moore swab samples using skim milk workflow as a whole, and (d) determine the limit of detection of SARS-CoV-2 in 10 ml of wastewater using automated Nanotrap workflow. The Nanotrap particles were used in several wastewater surveillance studies because of numerous advantages. This study will address whether Nanotrap workflows could substitute the traditional membrane filtration and skim milk workflows for viral concentration in wastewater and whether the Nanotrap workflows compromise the sensitivity of SARS-CoV-2 detection. In addition, we determined the limit of detection of automated Nanotrap workflow when a known amount of SARS-CoV-2 genome copies was spiked into 10 ml of wastewater.

## Materials and methods

### Wastewater samples

For this study, wastewater samples for method comparison were collected weekly from community manholes and influent lines of wastewater treatment plants, which represented tens of thousands of people who contributed to the collected sample in Atlanta from June 2021 to August 2021. In total, 500 ml of wastewater was collected in a sterile, autoclavable, polypropylene bottle. Wastewater samples were transported to the laboratory in a cooler with ice. A wastewater sample was collected from a residential septic tank from a vacation house, which was used to determine the limit of detection (LOD) of SARS-CoV-2. Before this sample collection, we confirmed that the residents in this house had no previous COVID-19 infections, and the wastewater was tested negative for SARS-CoV-2 prior to the LOD work. Wastewater samples from community manholes were also collected weekly using Moore swab, a passive sampling method, which was made from cotton gauze and secured by tying a fishing line to a hook at the top of the manhole, and the swabs were placed in wastewater flows for 24 h and then retrieved.

### Membrane filtration workflow

**Figure 1:**
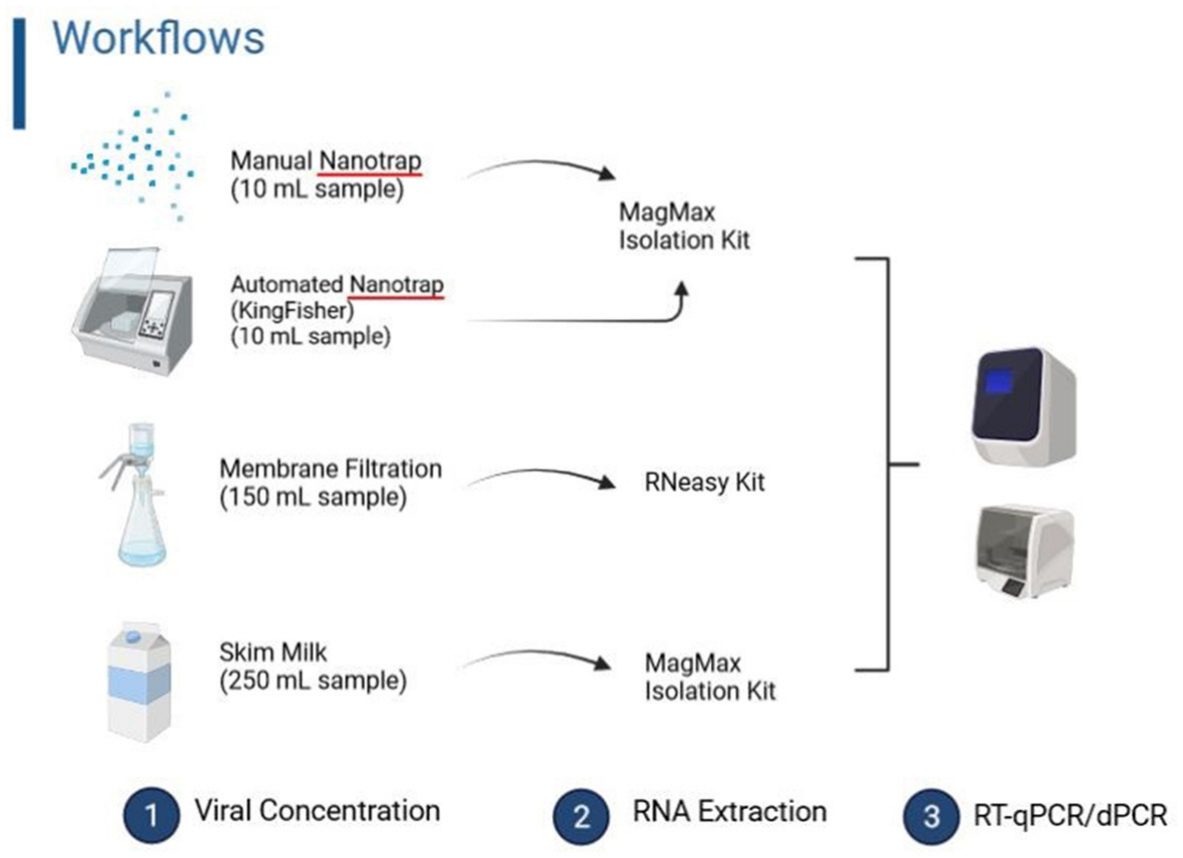
Four workflows (manual Nanotrap, automated Nanotrap, membrane filtration, and skim milk workflows) evaluated in this study.

Grab samples were centrifuged at 5,000 rpm for 5 min at 4°C to remove solids from the sample that could clog the membrane filter. The method using 0.45-μm-pore-size and 47-mm-diameter nitrocellulose filters, described by Liu et al. (2022), was used to concentrate SARS-CoV-2 from 150 ml of wastewater grab samples after pH adjustment to 3.5, and 25 mM of magnesium chloride was added. Before filtration, 10^5^ equivalent genome copies (GC) of bovine respiratory syncytial virus (BRSV) (INFORCE 3, Zoetis, Parsippany, NJ) were added to the sample as a process control. BRSV is a major cause of respiratory disease and a major contributor to the bovine respiratory disease (BRD) complex in cattle, particularly calves. It is a negative-sense, single-stranded RNA virus closely related to human respiratory syncytial virus (HRSV). Because BRSV does not exist in wastewater, the authors and other researchers successfully used this virus as a sample processing control to monitor the virus concentration, RNA extraction, and RT-qPCR procedures of SARS-COV-2 detection in wastewater. After filtration of wastewater, the membrane filter was placed into a microcentrifuge tube, and 800 μL of RLT buffer from the RNeasy Mini Kit (QIAGEN, Hilden, Germany) was added immediately. The membrane was vortexed at maximum speed for 10 min and then subjected to RNA extraction as described in the instructions of the RNeasy Mini Kit (Figure 1). Finally, 60 μl of RNA was eluted from each column.

### Skim milk flocculation workflow

Skim milk workflow (Figure 1) was used for SARS-CoV-2 concentration in Moore swab samples described by Liu et al. (2022) because these samples had higher turbidity. In brief, a 5% (w/v) skim milk solution was prepared by dissolving 5 g of skim milk powder (BD, #232100, Sparks, MD) in 100 ml of distilled water. Before the flocculation step, the liquid sample was squeezed from a Moore swab, processed using BigMixer 400 CC (Interscience for Microbiology, France) at speed 2 for 30 s, and was adjusted to 3.5 using 6 N HCL. Then, skim milk was added to the final concentration of 1% in 250 ml of wastewater, followed by the addition of 10^5^ GC of BRSV and shaking for 2 h. After this step, the sample was centrifuged (12,000 × *g* for 30 min), followed by RNA extraction using the Applied Biosystems MagMax^TM^ nucleic acid isolation kit (Thermo Fisher Scientific #48310). Finally, 60 μl of RNA was achieved from the extraction procedure.

### Manual nanotrap particles workflow

Nanotrap Microbiome A Particles (SKU#44202, Ceres Nanosciences Inc., Manassas, VA) are magnetically functionalized, affinity-capture hydrogel particles that capture and concentrate microbes from samples. The grab wastewater samples and the liquid squeezed from Moore swab samples were used for the manual Nanotrap workflow (Figure 1). In total, 150 μl of Nanotrap^®^ particles and 10 μl of BRSV, approximately equivalent to 10^5^ GC of BRSV, were added to 10 ml of wastewater. Seeded samples were incubated for 20 min and placed on a magnet rack (Thermo Fisher Scientific, Waltham, MA) for 10 min. The supernatant was then removed without disturbing the pellet of Nanotrap^®^ particles. One milliliter of molecular-grade water was used to rinse the pellet off the side of the tube, and the sample was transferred to a 1.7-ml tube. The sample was, then, placed on a small magnet rack for 2 min to allow the particles to pellet, followed by adding 140 μl of 1 × PBS to the particle pellet that was used for RNA extraction using the Applied Biosystems MagMax^TM^ nucleic acid isolation kit in accordance with the manufacturer's instructions. Finally, 60 μl of RNA was eluted from each sample.

### Automated nanotrap particle workflow

The KingFisher™ Apex robot platform (Thermo Fisher Scientific, USA), which allows 24 samples to be processed using a 24-plex head, was used for virus concentration and nucleic acid extraction from wastewater. In brief, 10 μl of BRSV (equivalent to 10^5^ GC of BRSV) and 50 μl of Nanotrap^®^ enhancement reagent 1 (ER1, Ceres Nanoscience Inc., #10111) were mixed with ~5 ml of wastewater, and two replicate wells with a total of 10 ml of wastewater were used for each sample. After 10 min of incubation at room temperature, 75 μl of Nanotrap^®^ particles were added, and the sample plates were loaded into the KingFisher™ Apex, followed by running the designated KingFisher™ script. After viral concentration, the samples were processed for RNA extraction using the Applied Biosystems MagMax^TM^ nucleic acid isolation kit on the same platform, following the manufacturer's instructions (Figure 1). Approximately 60 μl of RNA was achieved from each sample.

### Quantitative real-time RT-PCR method

SARS-CoV-2 RNA was detected via RT-qPCR (https://www.protocols.io/view/singleplex-qpcr-for-sars-cov-2-n1-and-brsv-b2qyqdxw) using the N1 primers described before (Lu et al., 2020; Liu et al., 2022) and the TaqPath^TM^ qPCR Master Mix (ThermoFisher Scientific, Waltham, MA). Synthetic SARS-CoV-2 RNA (ATCC, #VR-3276SD, Manassas, VA) with known concentration was 10-fold diluted, and the standard curve was used for quantification of SARS-CoV-2 RNA titers in wastewater. A diluted synthetic SARS-CoV-2 RNA served as a positive control and was included in each RT-qPCR run. Consistent Ct values from this positive control [mean = 24.1; standard deviation (SD) = 0.51] were observed during the study period. All the samples were tested in duplicates, and 5 μl of RNA was used for each PCR reaction. Potential PCR inhibition was examined in some samples through testing dilutions (1:5 and 1:10) and comparing the results with those from the undiluted RNA. Positive samples were defined as the presence of Ct values in both duplicate wells from one sample.

### dPCR RT-PCR method

Digital PCR was performed using the QIAcuity digital PCR system (Qiagen, Hilden, Germany). SARS-CoV-2 RNA was detected using QIAcuity one-step viral RT-PCR kit (Qiagen, catalog #1123145) following the manufacturer's protocol. The QIAcuity instrument was configured with the following parameters: reverse transcription at 50°C for 40 min, PCR initial heat activation with a single cycle at 95°C for 2 min, and PCR cycling with 45 cycles at 95°C for 5 s, followed by annealing/extension at 50°C for 30 s.

### Limit of detection of automated nanotrap workflows

Inactivated SARS-CoV-2 (ATCC, 2019-nCoV/USA-WA1/2020) with a known concentration in GC/μl was 10-fold serially diluted and spiked into 10 ml of wastewater collected from the septic tank wastewater sample. The wastewater, collected from a septic tank where no family members had been infected with COVID-19 before, was tested SARS-CoV-2-negative using our in-house Nanotrap workflow and RT-qPCR. The SARS-CoV-2 serially diluted samples were processed using the aforementioned automated Nanotrap workflow, and SARS-CoV-2 RNA was detected using both RT-qPCR and dPCR methods. This experiment was performed three times, and the limit of detection for the Nanotrap workflow was determined that the most diluted SARS-CoV-2 was detected in all three experiments. The average Ct value and positive partitions were calculated from the three experiments.

### Statistical analysis

Two-group *t*-test was used to compare Ct values between the automated and manual Nanotrap workflows with and without ER1 in 10 ml of grab and Moore swab samples, between the automated Nanotrap workflow and the membrane filtration workflow in grab samples, and between the automated Nanotrap workflow and the skim milk workflow in Moore swab samples. Differences were considered statistically significant if the *p*-value was < 0.05.

## Results

### Effectiveness of enhancement reagent 1

**Figure 2:**
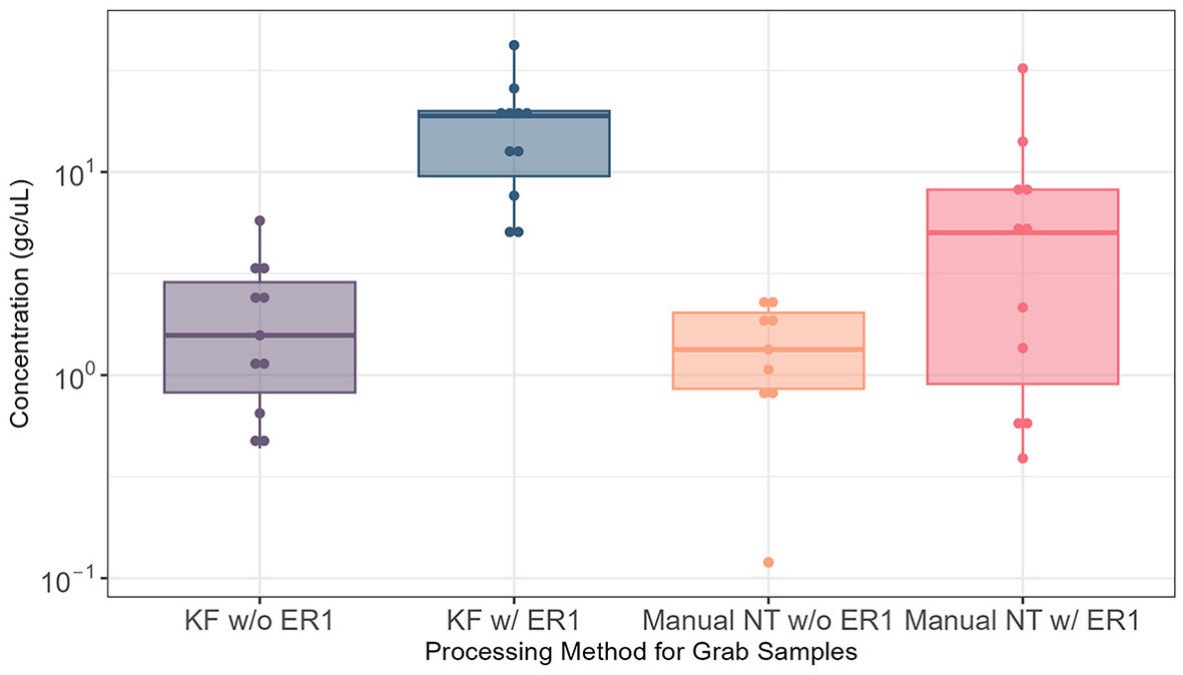
Comparison of SARS-CoV-2 RNA detection in 10 ml of grab wastewater samples from influent lines processed using the automated and manual Nanotrap workflows with and without ER1. KF w/o ER1, KingFisher (automated) Nanotrap particles workflow without enhancement reagent 1; KF w/ER1, KingFisher with enhancement reagent 1; Manual NT w/o ER1, manual Nanotrap particles workflow without enhancement reagent 1; Manual NT w/ER1, manual Nanotrap particle workflow with enhancement reagent 1. *Y*-axis: GC/uL, genome copies per microliter of RNA.

The effectiveness of ER1 for the Nanotrap particles was examined by comparing the automated and manual Nanotrap workflows with and without ER1 in 10 ml wastewater using grab samples (*N* = 22) from influent lines. Adding ER1 to 10 ml of grab wastewater prior to viral concentration on the KingFisher system (automated) resulted in significantly higher (*P* = 0.0005) viral concentration compared with the protocol without ER1. When the manual Nanotrap workflow was used, adding ER1 to 10 ml of grab samples also showed a similar improvement in viral concentration (Figure 2). These results suggest that including ER1 in the viral concentration significantly increases the viral detection in grab samples using both the manual and automated Nanotrap workflows.

**Figure 3:**
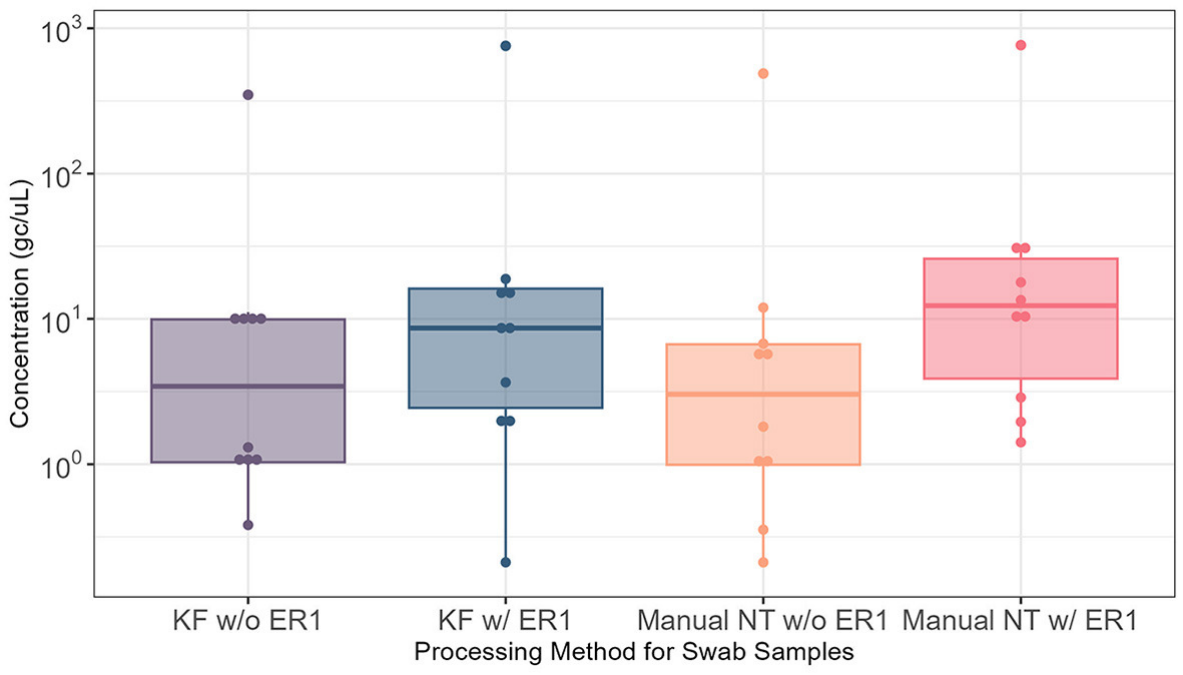
Comparison of SARS-CoV-2 RNA detection in 10 ml of Moore swab samples processed using the KingFisher automated and manual Nanotrap workflows with and without adding ER1. KF w/o ER1, KingFisher (automated) Nanotrap particles workflow without enhancement reagent 1; KF w/ER1, KingFisher with enhancement reagent 1; Manual NT w/o ER1, manual Nanotrap particles workflow without enhancement reagent 1; Manual NT w/ER1, manual Nanotrap particle workflow with enhancement reagent 1. *Y*-axis: GC/uL, genome copies per microliter of RNA.

We then examined the effect of ER1 using Moore swab samples (*N* = 14) and again compared the automated and manual Nanotrap workflows in 10 ml of samples with and without ER1. Adding ER1 to the 10 ml of liquid squeezed from the Moore swab samples prior to viral concentration with Nanotrap particles yielded significantly higher viral concentration (*P* < 0.05) in the automated Nanotrap workflow. Adding ER1 to 10 ml of Moore swab samples using the manual Nanotrap workflow also demonstrated higher viral concentration compared with samples without ER1, but the viral concentration from the Moore swab samples was more variable than the grab samples (Figure 3). These results indicate that ER1 significantly increases the viral concentration in the Moore swab samples using both the manual and automated Nanotrap workflows.

### Comparison of automated nanotrap workflow and membrane filtration workflow

To validate whether the Nanotrap workflow can achieve comparable results to the membrane filtration workflow, we performed a side-by-side comparison of the automated Nanotrap workflow with ER1 in 10 ml of wastewater samples and the membrane filtration workflow in 150 ml of grab samples (Table 1). A total of 15 wastewater grab samples were processed in parallel using the automated Nanotrap workflow and the membrane filtration workflow. In comparison to membrane filtration workflow, automated workflow with Nanotrap particles achieved an average of 63.8 genome copies per microliter of extracted SARS-CoV-2 RNA in 15 wastewater samples, significantly higher (*P* = 0.0083) than an average of 27.8 genome copies per microliter of extracted RNA in 150 mL of wastewater using membrane filtration workflow, and BRSV also showed the same trend (Table 1). These results suggest that using Nanotrap particles with ER1 to concentrate viruses from a small volume of grab samples can achieve better results than using membrane filtration workflow in a large volume of wastewater.

**Table 1:** Comparison of SARS-CoV-2 and BRSV detection using the automated Nanotrap workflow with ER1 vs. membrane filtration workflow in wastewater.

**Workflow**	**No. sample**	**Positive (%)**	**N1 Ct (SD)**	**Average N1 GC/μL (SD)^‡^**	**BRSV Ct (SD)**
Automated Nanotrap with ER1 (10 ml)	15	15 (100)	31.55 (1.15)	63.8 (8.7)	26.73 (1.26)
Membrane Filtration (150 ml)	15	15 (100)	32.78 (1.96)	27.8 (15.8)	32.55 (3.06)

SD, standard deviation.

^‡^Average SARS-CoV-2 genome copies per microliter of RNA extracted.

Wilcoxon Signed Rank Test: automated Nanotrap workflow with ER1 vs. membrane filtration workflow, *P* = 0.0083 (N1) and *P* < 0.0001 (BRSV).

Because we concentrated viruses in 150 ml of wastewater and there was a possibility that PCR inhibitors were concentrated simultaneously, which may impact PCR results, a subset of paired RNA samples (Nanotrap vs. membrane filtration) was tested for PCR inhibition. The RNA sample was 1:5 and 1:10 diluted. Compared with the undiluted RNA, 1:5 and 1:10 diluted RNA samples showed increased Ct values for both SARS-CoV-2 N1 and BRSV (data not shown). These results indicate that the grab samples we collected in Atlanta areas have no PCR inhibition.

### Comparison of automated nanotrap workflow and skim milk workflow

Similarly, wastewater extracted from 29 Moore swab samples was processed side-by-side using the automated Nanotrap workflow, with ER1 in 10 ml of samples and the skim milk workflow in 250 ml of samples. The average N1 and BRSV Ct values for the 10 ml of samples processed using the Nanotrap particles were 31.92 (an average of 50.4 SARS-CoV-2 genome copies per microliter of extracted RNA) and 27.92, significantly better (*P* = 0.006 for N1 Ct comparison) than 32.97 (an average of 24.0 SARS-CoV-2 genome copies per microliter of extracted RNA) and 34.44, the average N1 and BRSV Ct values for the skim milk workflow using 250 ml of samples (Table 2), respectively. These results indicate that using Nanotrap particles to concentrate viruses from a small volume of Moore swab samples can achieve significantly better results than skim milk workflow in large-volume swab samples.

**Table 2:** Comparison of SARS-CoV-2 and BRSV detection using the automated Nanotrap particle and skim milk workflows in wastewater.

**Workflow**	**Sample number**	**Positive (%)**	**N1 Ct (SD)**	**Average N1 GC/μL (SD)^‡^**	**BRSV Ct (SD)**
Automated Nanotrap with ER1 (10 ml)	29	29 (100)	31.92 (1.60)	50.4 (15.4)	27.92 (0.98)
Skim milk (250 ml)	29	29 (100)	32.97 (1.58)	24.0 (12.3)	34.44 (1.66)

SD, standard deviation.

^‡^Average SARS-CoV-2 genome copies per microliter of RNA extracted.

Wilcoxon Signed Rank Test: automated Nanotrap workflow with ER1 vs. skim milk workflow, *P* = 0.006 (N1) and *P* < 0.0001(BRSV).

Similarly, a subset of paired RNA samples from Nanotrap and membrane filtration concentration methods was tested for PCR inhibition in the same way described above. Compared with the undiluted RNA, 1:5 and 1:10 diluted RNA samples showed increased Ct values for both SARS-CoV-2 N1 and BRSV (data not shown). These results suggest that the Moore swab samples have no PCR inhibition.

### Limit of detection of the automated nanotrap workflow

Table 3 shows the limit of detection (LOD) of SARS-CoV-2 in 10 ml of grab wastewater using the automated Nanotrap workflow and the RT-qPCR/dPCR methods. The experiments were performed three times. LOD was defined as the detection of the lowest spiked amount of SARS-CoV-2 in 10 ml of wastewater in all three replicated experiments. Inactivated SARS-CoV-2 was 10-fold serially diluted, and the diluted SARS-CoV-2 was spiked into 10 ml of wastewater at concentrations (genome copies) of 1.15 × 10^4^/ml, 1.15 × 10^3^/ml, 1.15 × 10^2^/ml, 11.5/ml, and 1.5/ml, followed by automated Nanotrap workflow for viral concentration and RNA extraction. When RT-qPCR was used, 11.5 GC/ml spiking level (115 GC in total) was the limit of detection in 10 ml of wastewater while 115 GC/ml spiking level (1,150 GC in total) was the limit of detection when dPCR was used.

**Table 3:** SARS-CoV-2 limit of detection in 10 ml of wastewater using automated Nanotrap workflow.

**Spiking genome copies/ml**	**RT-qPCR**	**dPCR**
	**Average Ct (SD)** ^*^	**Average positive partition (SD)** ^*^
1.15 × 10^4^	29.4 (0.7)	1,046.5 (201.6)
1.15 × 10^3^	32.5 (0.8)	110.8 (32.3)
1.15 × 10^2^	36.4 (0.8)	9.5 (3.1)
1.15 × 10	40.5 (1.2)	0.5 (0.6)
1.15	Not detected	Not detected

^*^Average from three experiments.

## Discussion

Since the COVID-19 pandemic, many wastewater-based epidemiology studies have focused on the detection of SARS-CoV-2 RNA in wastewater collected from wastewater treatment plants, community manholes, campus residence halls, and buildings (Ahmed et al., 2020; Wu et al., 2020, 2021; Khan et al., 2021; Liu et al., 2022). Although infected human individuals shed a relatively high titer of SARS-CoV-2 in their feces, viruses can become highly diluted when the virus eventually enters the sewage system and are generally present in low concentrations in wastewater (Yang et al., 2022), especially when case numbers are lower in communities. To detect SARS-CoV-2 RNA in wastewater, researchers tend to concentrate viruses from large volumes (Barril et al., 2021; Fores et al., 2021; Philo et al., 2022a) into smaller volumes or precipitate the viruses into a pellet (Wolfe et al., 2021) so that nucleic acid extraction and molecular detection methods can be applied. For this purpose, most studies apply one, e.g., skim milk method alone or two in-series methods, e.g., starting ultrafiltration method from a large volume followed by skim milk further concentration to a small volume. Another important concern when using molecular methods to detect viral RNA in wastewater is that certain organic matter and chemicals can inhibit RT-PCR reactions and can be concentrated along with SARS-CoV-2. These inhibitors can cause a weaker PCR signal or even false negative results. Due to these limitations, there is a need for a simple, rapid, robust, and efficient concentration method that can be automated for large-scale COVID-19 wastewater surveillance or performed manually in resource-limited areas.

In this study, we utilized a Nanotrap particle workflow for virus concentration in wastewater. Our results indicated that adding ER1 to wastewater prior to viral concentration using the Nanotrap particles significantly improved PCR results in 10 ml of samples processed either in automated or manual workflow compared with the same method without ER1. We noted an increasing N1 Ct value by 4.05 (SD = 1.13) for the grab samples and 2.25 (SD = 1.60) for the swab samples compared with the same sample and the same method without ER1. In addition, SARS-CoV-2 RNA detection in 10 ml of grab samples with Nanotrap particles and ER1 showed significantly better results than 150 ml of Moore grab samples using the membrane filtration workflow and 250 ml of Moore swab samples using the skim milk workflow. We believe that this effect is derived from the robustness of Nanotrap particles and ER1.

The limit of detection of SARS-CoV-2 in wastewater mainly depends on three lab procedures as follows: virus concentration, RNA extraction, and RT-qPCR/dPCR. On average, the lowest titers of SARS-CoV-2 RNA were quantified at 10−3.19 × 10^3^ GC/mL in wastewater (Gonzalez et al., 2020; Cervantes-Aviles et al., 2021). A similar range of the SARS-CoV-2 detection limit was determined by spiking known genome copies of SARS-CoV-2 into negative wastewater (Perez-Cataluna et al., 2021). By using the latter strategy to determine the LOD, there needs to be some caution since a trace amount of SARS-CoV-2 may exist in what we tested as negative wastewater that we used for the spiking experiment, which may underestimate the LOD of our methods. In our study, the LOD, ranging from 11.5 GC/ml using RT-qPCR as a testing method to 115 GC/ml using dPCR as a detection method, was determined, and this LOD is consistent with previously reported results (Gonzalez et al., 2020; Cervantes-Aviles et al., 2021; Perez-Cataluna et al., 2021).

When the direct cost, including concentration reagents and supplies, consumables, and RNA extraction kit, of the four workflows (Nanotrap automated, Nanotrap manual, skim milk, and membrane filtration) is compared, skim milk workflow turns out to be the most affordable workflow. However, when the indirect cost, e.g., processing time, equipment requirement, and sample volume (how easy to collect sample), is included, the Nanotrap particle manual method tends to be the most convenient and easy workflow among the four workflows, although the direct cost of the Nanotrap manual workflow is slightly higher than skim milk and membrane filtration workflows (Table 4). Compared with skim milk and membrane filtration workflows, concentrating viruses from wastewater using Nanotrap particles has several advantages as follows: (1) small sample volume (10 ml), which is easier to collect and transport; (2) simple equipment, only requiring a magnetic tube rack, which is appropriate for low-resource settings; (3) potential to adapt to high-throughput platform in high resource settings for scalable implementation (Lin et al., 2020; Karthikeyan et al., 2021a); (4) more sensitive than traditional large volume concentration methods (membrane filtration and skim milk workflows); and (5) rapid–viral concentration takes significantly less than an hour and requires no additional centrifugation or filtration steps for both the high throughput and manual workflows; (6) long shelf life of Nanotrap particles which makes easy for storage and transportation. All of these advantages enable this method to be used in resource-limited areas if no appropriate centrifuge is available. If centrifuge is available, skim milk would be a priority to be considered. In areas without resource limitation, SARS-CoV-2 wastewater surveillance could be implemented via the automated Nanotrap approach.

**Table 4:** Comparison of Nanotrap Microbiome A Particles, skim milk, and membrane filtration workflows for SARS-CoV-2 detection in wastewater.

	**Nanotrap Microbiome A Particles workflow**		**Skim milk workflow**	**Membrane filtration Workflow**
	**Automated**	**Manual**		
Cost ($)/per sample^*^	14.8	12.0	8.3	10.0
Processing Time (hrs) for 10 samples^*^	2.5	3.0–3.5	4.0–5.0	4.0–6.0
Sample volume (mL)	10	10 or 40	250	150
Sample type	Grab or swab	Grab or swab	Better for swab samples	Better for grab samples
Advantage	- Automatic - Small sample volume - Sensitive - Can be used on turbid and clear water samples - MagMax kit is cheaper than RNeasy mini kit - Can be implemented at large scale	- Easy to use and not a long protocol - Stable to store and transport - Small volume of sample for high sensitivity - Can be used on turbid and clear water samples - Only a microcentrifuge required	- Affordable - Low number of consumables used	- Affordable - Only a microcentrifuge required - Low number of consumables used
Disadvantage	- Expensive equipment - More plastic consumable	- Requires magnetic separator - Nanotrap particles are expensive than skim milk and membrane - Can not be scalable	- Requires centrifuge if large volume is used - More time-consuming for flocculation - Not sensitive compared with the Nanotrap method - Can not be scalable	- Filtration may take more time if water sample is turbid - Not sensitive compared with the Nanotrap method - RNeasy Mini kit is expensive than MagMax - Can not be scalable

^*^The cost includes virus concentration reagents, consumables, and RNA extraction kit.

## Data availability statement

All relevant data presented in the study are included in the article/Supplementary material. Further inquiries can be directed to the corresponding author.

## Author contributions

PL wrote the manuscript and contributed to the design and conception of the study. PL, BL, RBarb, and CM acquired the funding and revised the manuscript. MW and RBarc contributed to the design and conception of the study. LG, MC, CC, AN, AL, JD, and OS performed the laboratory work. SH performed the statistical analysis. All authors contributed to the article and approved the submitted version.
