# Supplementary material for: Comparison of Nanotrap® Microbiome A Particles, membrane filtration, and skim milk workflows for SARS-CoV-2 concentration in wastewater
Source: Front Microbiol. 2023 Jul 5;14:1215311. doi: 10.3389/fmicb.2023.1215311 (PMC10354513; doi:10.3389/fmicb.2023.1215311)
Supplement: Supplementary file 1 [file Table_1.docx]

Supplementary Table 1. Summary of RT-qPCR results of three RNA extraction kits for SARS-CoV-2 and BRSV (spiking control) detection in 10 mL wastewater

| **No. of Data Points** | **Extraction Kit Used** | **Average N1 CT Value (SD)** | **Average BRSV CT Value (SD)** |
| --- | --- | --- | --- |
| 16 | RNeasy Mini | 35.81 (1.31) | 26.83 (0.42) |
| 16 | MagMax | 36.25 (1.12) | 27.59 (0.32) |
| 16 | IDEXX | 36.40 (1.50) | 27.85 (0.30) |
