# Supplementary material for: Comparison of Nanotrap® Microbiome A Particles, membrane filtration, and skim milk workflows for SARS-CoV-2 concentration in wastewater
Source: Front Microbiol. 2023 Jul 5;14:1215311. doi: 10.3389/fmicb.2023.1215311 (PMC10354513; doi:10.3389/fmicb.2023.1215311)
Supplement: Supplementary file 2 [file Table_2.docx]

Supplementary Table 2. Summary of BRSV recovery in 150 mL wastewater using membrane filtration for viral concentration, RNeasy mini kit for RNA extraction, and dPCR for detection

| Wastewater  Sample No. | Wastewater Volume (mL) | Concentration Method | RNA Extraction Method | BRSV Spiking Level (Total GC) | BRSV Recovered (Total GC)^‡^ | Recovery Efficiency (%) |
| --- | --- | --- | --- | --- | --- | --- |
| 1 | 150 | MF* | RNeasy Kit | 4,843,442 | 65,568 | 1.35 |
| 2 | 150 | MF | RNeasy Kit | 4,843,442 | 210,864 | 4.35 |
| 3 | 150 | MF | RNeasy Kit | 4,843,442 | 93,984 | 1.94 |
| 4 | 150 | MF | RNeasy Kit | 4,843,442 | 12,201 | 0.25 |
| 5 | 150 | MF | RNeasy Kit | 4,843,442 | 15,526 | 0.32 |

*Membrane filtration; ^‡^detected by dPCR

GC: genome copies
