# Supplementary material for: Comparison of Nanotrap® Microbiome A Particles, membrane filtration, and skim milk workflows for SARS-CoV-2 concentration in wastewater
Source: Front Microbiol. 2023 Jul 5;14:1215311. doi: 10.3389/fmicb.2023.1215311 (PMC10354513; doi:10.3389/fmicb.2023.1215311)
Supplement: Supplementary file 3 [file Table_3.docx]

Supplementary Table 3. Summary of BRSV recovery in 10 mL wastewater using Nanotrap particles for viral concentration, MagMax kit for RNA extraction, and dPCR for detection

| Wastewater  Sample No. | Wastewater Volume (mL) | Concentration Method | RNA Extraction Method | BRSV Spiking Level (Total GC) | BRSV Recovered (Total GC)^‡^ | Recovery Efficiency (%) |
| --- | --- | --- | --- | --- | --- | --- |
| 1 | 10 | NP | MagMax | 4,843,442 | 3,345,096 | 69.06 |
| 2 | 10 | NP | MagMax | 4,843,442 | 3,555,648 | 73.41 |
| 3 | 10 | NP | MagMax | 4,843,442 | 3,780,000 | 78.04 |
| 4 | 10 | NP | MagMax | 4,843,442 | 4,346,400 | 89.73 |

*Nanotrap particles; ^‡^detected by dPCR

GC: genome copies
